# Higher PGD_2_ production by synovial mast cells from rheumatoid arthritis patients compared with osteoarthritis patients via miR-199a-3p/prostaglandin synthetase 2 axis

**DOI:** 10.1038/s41598-021-84963-7

**Published:** 2021-03-11

**Authors:** Shintaro Mishima, Jun-ichi Kashiwakura, Shota Toyoshima, Tomomi Sasaki-Sakamoto, Yutaka Sano, Kazuyoshi Nakanishi, Kenji Matsumoto, Yoshimichi Okayama

**Affiliations:** 1 Allergy and Immunology Research Project Team, Nihon University School of Medicine, 30-1 Oyaguchi-kamicho, Itabashi-ku, Tokyo, 173-8610 Japan; 2 Department of Orthopaedic Surgery, Nihon University School of Medicine, Tokyo, Japan; 3 Department of Immunology, Graduate School of Pharmaceutical Sciences, Hokkaido University, Sapporo, Japan; 4 Center for Medical Education, Nihon University School of Medicine, Tokyo, Japan; 5 Center for Allergy, Nihon University Itabashi Hospital, Tokyo, Japan; 6 Department of Allergy and Clinical Immunology, National Research Institute for Child Health and Development, Tokyo, Japan

**Keywords:** Osteoarthritis, Rheumatoid arthritis

## Abstract

We previously reported that synovial mast cells (MCs) from patients with rheumatoid arthritis (RA) produced TNF-α in response to immune complexes via FcγRI and FcγRIIA. However, the specific functions of synovial MCs in RA remain unclear. This study aimed to elucidate those functions. Synovial tissues and fluid were obtained from RA and osteoarthritis (OA) patients undergoing joint replacement surgery. Synovium-derived, cultured MCs were generated by culturing dispersed synovial cells with stem cell factor. We performed microarray-based screening of mRNA and microRNA (miRNA), followed by quantitative RT-PCR-based verification. Synovial MCs from RA patients showed significantly higher prostaglandin systhetase (PTGS)1 and PTGS2 expression compared with OA patients’ MCs, and they produced significantly more prostaglandin D_2_ (PGD_2_) following aggregation of FcγRI. PGD_2_ induced IL-8 production by human group 2 innate lymphoid cells, suggesting that PGD_2_-producing MCs induce neutrophil recruitment into the synovium of RA patients. PTGS2 mRNA expression in RA patients’ MCs correlated inversely with miRNA-199a-3p expression, which down-regulated PTGS2. RA patients’ synovial fluid contained significantly more PGD_2_ compared with OA patients’ fluid. Synovial MCs might regulate inflammation in RA through hyper-production of PGD_2_ following FcRγ aggregation. Our findings indicate functional heterogeneity of human MCs among diseases.

## Introduction

Rheumatoid arthritis (RA) is a chronic systemic inflammatory disease characterized by immune cell-mediated destruction of the architecture of joints. Proinflammatory cytokines, such as tumor necrosis factor (TNF)-α, interleukin (IL)-1β, IL-6 and IL-17, are thought to play pivotal roles in that joint architecture destruction in RA patients. In RA patients, the number of degranulated mast cells (MCs) is increased in synovial tissues and correlates with the disease activity^1–4^. Such MC mediators as histamine and tryptase in synovial fluid (SF) are also increased in these patients^2,4–6^. We previously reported that aggregated IgG induces TNF-α production by human synovium-derived, cultured MCs via FcγRI and FcγRII^7^ and that IL-33 synergistically enhances immune complex-induced TNF-α and IL-8 production in those same cells^8^. These results suggest that activation of MCs may exaggerate inflammation in RA. On the other hand, IL-33- and immune complex-triggered activation of human peripheral blood-derived, cultured MCs reportedly down-regulated monocyte-mediated immune responses via IL-10 and histamine release^9^, suggesting that MCs might have anti-inflammatory functions. The mRNA levels of MC-specific genes were reportedly inversely associated with disease severity in RA patients^9^. C-reactive protein and the tryptase serum level were negatively correlated in RA patients^10^, Thus, MCs might have both beneficial and harmful roles in inflammation, depending on their site and time of action and the etiology of the inflammatory response.

We previously compared the expression levels of Fc receptors and protease phenotypes of synovial MCs from RA and osteoarthritis (OA) patients and found no significant differences between the two groups^7^. Thus, the specific function or functions of synovial MCs of RA patients have not yet been elucidated. We hypothesized that RA patients’ synovial MCs have special characteristics and play specific roles in the inflammatory response in RA.

Upregulation of the cyclooxygenase and lipoxygenase pathways of arachidonic acid is thought to be involved in the development of rheumatic diseases, and targeting this pathway might lead to improved treatment strategies^11^. Indeed, prostaglandins (PGs) such as PGE_2_ and PGI_2_, and leukotrienes (LTs) such as LTB_4_, have been considered to play important roles in the onset and development of arthritic diseases in animals and humans^11–14^. However, some PGs, such as the nonenzymatic PGD_2_ metabolite 15-deoxy-PGJ_2_, have anti-inflammatory effects depending on the disease context^11^. PGD_2_ may exert pro-inflammatory or anti-inflammatory effects in different biologic systems^15^. PGD_2_ is a ligand for two receptors, D prostanoid (DP) and chemoattractant receptor-homologous molecule expressed on Th2 cells (CRTH2)^15^. PGH_2_ is converted to PGD_2_ by two enzymes, hematopoietic PGD synthase (HPGDS) and lipocalin-type PGDS (LPGDS). HPGDS is present in hematopoietic cells, including MCs^16^, and also in antigen-presenting cells^17^ and T helper 2 (Th2) lymphocytes^18^. On the other hand, LPGDS is present mainly in neuronal cells, but also in macrophages in pathologic situations^15^. PGD_2_ is produced in articular tissues of mice during the development of collagen-induced arthritis and plays an anti-inflammatory role, acting through the DP receptor^19^. CRTH2 deficiency was reported to exacerbate complete Freund’s adjuvant (CFA)-induced joint inflammation by attenuating infiltration of macrophages^20^. HPGDS-derived PGD_2_ was reported to ameliorate joint inflammation by attenuating vascular permeability and subsequent angiogenesis, and daily administration of a DP agonist attenuated CFA-induced hyperpermeability and angiogenesis^21^. A clinical study found high concentrations of PGD_2_ in the SF of patients with RA, and PGD_2_ and HPGDS expressions were inversely associated with serum C-reactive protein (*P* < 0.01)^22^. These findings suggest that PGD_2_ is an active agent in the resolution of inflammation in experimentally induced murine arthritis and in human RA.

In this study, we show that human synovial MCs from RA patients produced greater amounts of PGD_2_ via the PTGS2-miR-199a-3p axis compared with MCs from OA patients. Elevated PGD_2_ production in RA patients’ MCs might play an important role in the pathogenesis of RA.

## Results

### Comparison of gene expression profiles between synovial MCs from OA and RA patients

**Figure 1:**
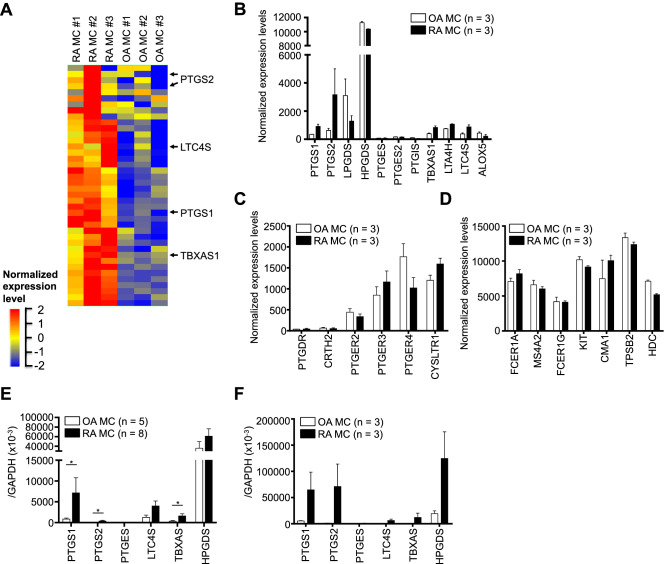
Comparison of gene expression profiles between synovial MCs from OA and RA patients. (**A**) A part of hierarchical clustering on the basis of the gene expression data in synovium-derived, cultured MCs obtained from three RA patients compared with MCs from three OA patients. Total RNA was isolated from the cultured MCs without any stimulus. Each row of colored bars represents one gene, and each column represents one donor. Colored bars show the magnitude of the response of each gene, according to the scale shown. Arrows indicate genes that are associated with enzymes involved in eicosanoid biosynthesis. (**B**–**D**) Comparison of gene expression levels of enzymes involved in eicosanoid biosynthesis (**B**), receptors for eicosanoid (**C**) and MC-related molecules (**D**) in synovium-derived, cultured MCs from three OA patients (open bars) and three RA patients (closed bars). Data are shown as normalized expression levels of DNA chip (mean ± SEM). (**E**) Comparison of gene expression levels of enzymes involved in eicosanoid biosynthesis in synovium-derived, cultured MCs obtained from OA (open bars, n = 5) and RA (closed bars, n = 8) patients. The data are shown as the ratio of the enzyme mRNA level to the GAPDH mRNA level (mean ± SEM). Significance was determined using the Mann–Whitney *U* test (*P < .05). (**F**) Comparison of gene expression levels of enzymes involved in eicosanoid biosynthesis in fleshly isolated, synovial MCs from OA (open bars, n = 3) and RA (closed bars, n = 3) patients.

We first compared the gene expression profiles between synovium-derived, cultured MCs from OA patients (n = 3 donors) and those from RA patients (n = 3 donors) using DNA chips. A total of 419 genes among approximately 42,545 full-length genes and expressed sequence tags were significantly more than two fold higher in synovium-derived, cultured MCs from RA patients than in OA patients. Figure 1A shows a part of hierarchical clustering on the basis of those gene expression data. Upregulation of the cyclooxygenase and lipoxygenase pathways of arachidonic acid is thought to be involved in the development of rheumatic diseases, and targeting these pathways might lead to improved treatment strategies^11^. For that reason, from among those upregulated genes, we focused on prostanoid synthetases, including prostaglandin synthetase (PTGS)1, PTGS2, thromboxane synthetase 1 (TBXAS1) and leukotriene C_4_ synthetase (LTC4S) (Fig. 1A). DNA chip analysis showed that the normalized expression level of HPGDS was high compared with the other prostanoid synthetases and did not differ significantly between MCs from OA and RA patients (Fig. 1B). The expression levels of PTGES, PTGES2 and PTGIS were quite low. The expression levels of prostanoid receptors (Fig. 1C) and MC-related genes (Fig. 1D) did not appear to differ significantly between MCs from OA and RA patients. We confirmed the above DNA chip results using quantitative RT-PCR (Fig. 1E,F). The expression levels of mRNA for PTGS1, PTGS2 and TBXAS1 were significantly higher in RA patients’ MCs than in OA patients’ MCs (Fig. 1E). The expression levels of mRNA for LTC4S showed the tendency of being high in RA patients’ MCs compared with OA patients’ MCs. Furthermore, we confirmed those findings in synovium-derived, cultured MCs by using freshly isolated synovial MCs from OA and RA patients (Fig. 1F).

### Comparison of histamine release and eicosanoid synthesis by synovium-derived, cultured MCs from OA and RA patients following FcR γ-chain cross-linking

**Figure 2:**
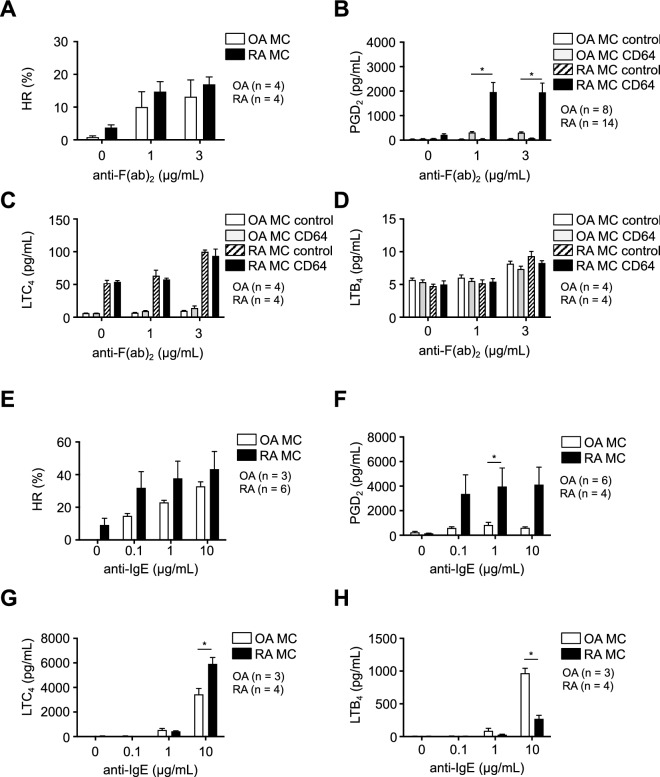
Comparison of histamine release (**A**,**E**) and PGD_2_ (**B**,**F**), LTC_4_ (**C**,**G**) and LTB_4_ synthesis (**D**,**H**) by synovium-derived, cultured MCs obtained from OA and RA patients following FcγRI aggregation (**A**–**D**) and FcεRI aggregation (**E**–**H**). Data are shown as % HR (histamine release) or pg/mL (/10^5^ cells, mean ± SEM). Significance was determined by Sidak’s-Bonferroni test (*P < .05).

Histamine release from synovium-derived, cultured MCs following FcγRI and FcεRI aggregation did not differ significantly between OA and RA patients (Fig. 2A,E), while PGD_2_ production by the RA patients’ MCs was significantly higher than by the OA patients’ MCs (Fig. 2B,F). Following FcγRI aggregation, MCs from both OA and RA patients produced very small amounts of LTC_4_ and LTB_4_ (Fig. 2C,D). Following FcεRI aggregation, LTC_4_ synthesis in RA patients’ MCs was significantly higher than in OA patients’ MCs (Fig. 2G), while LTB_4_ synthesis was significantly lower in RA patients’ MCs than in OA patients’ MCs (Fig. 2H).

### PGD_2_ induced IL-8 production by human group 2 innate lymphoid cells (ILC2s)

**Figure 3:**
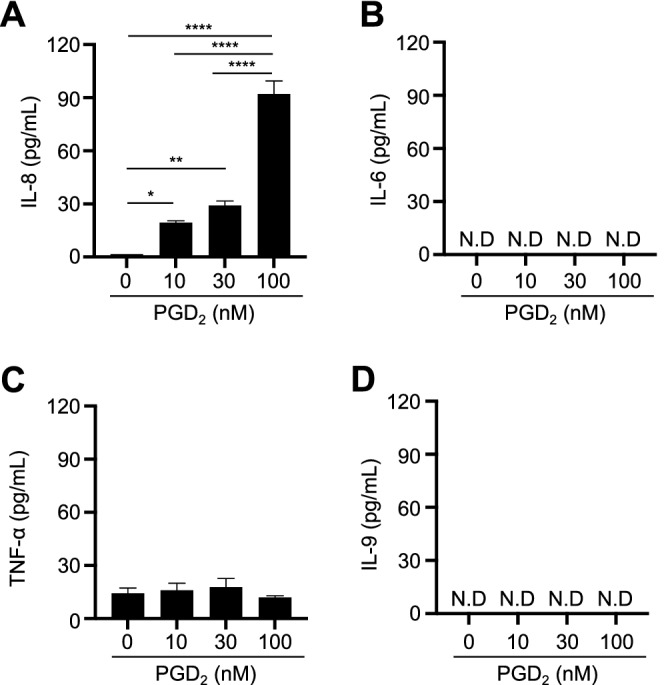
PGD_2_ induced IL-8 production by human group 2 innate lymphoid cells (ILC2s). IL-8 (**A**), IL-6 (**B**), TNF-α (**C**) and IL-9 production (**D**) by ILC2s in response to PGD_2_. The results are the mean ± SEM for three different donors. Significance was determined by Sidak’s-Bonferroni test (*P < .05, **P < .01, and ****P < .0001). N.D. means not detected (below the level of detection).

We investigated the pathogenic roles of PGD_2_-producing MCs in RA. We first investigated whether PGD_2_ induced IL-6, IL-8 and TNF-α production by human synovium-derived, cultured MCs, but found that it did not (data not shown). Group 2 innate lymphoid cells (ILC2s) were reported to be involved in the pathogenesis of RA^23,24^. MCs and ILC2s are located in close proximity in such tissues as the skin^25^ and lung^26^. ILC2s were found in the synovium of RA patients^23,24^. PGD_2_ induced production of Th2 cytokines such as IL-5 and IL-13 by ILC2s^27^. Thus, we investigated the effects of PGD_2_ on production of IL-6, IL-8, IL-9 and TNF-α by ILC2s. We found that PGD_2_ induced production of IL-8, but not IL-6 and IL-9, by ILC2s (Fig. 3A,B,D). TNF-α was spontaneously released by ILC2s and PGD_2_ did not enhance the production (Fig. 3C). This suggests that higher amounts of IL-8 produced by ILC2s in RA patients’ synovial tissues in response to IgG-mediated MC-induced PGD_2_ might cause neutrophil recruitment into the synovium of those patients.

### Effects of co-culture of OA patients’ synovial MCs with RA patients’ synovial fibroblasts on PTGS1, PTGS2, TBXAS1 and LTC4S expression in OA patients’ MCs

**Figure 4:**
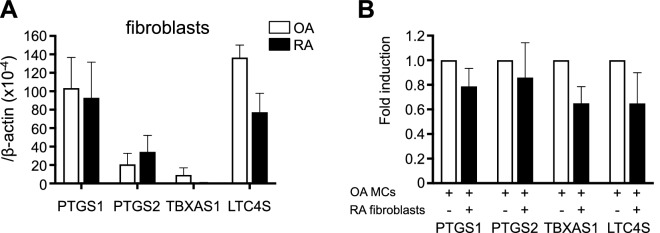
Effects of co-culture of OA patients’ synovial MCs with RA patients’ synovial fibroblasts on expression levels of mRNA for PTGS1, PTGS2, TBXAS1 and LTC4S in the OA patients’ MCs. (**A**) Comparison of expression levels of mRNA for PTGS1, PTGS2, TBXAS1 and LTC4S in fibroblasts obtained from OA (open bars, n = 4) and RA (closed bars, n = 4) patients. The data are shown as the ratio of the enzyme mRNA level to the β-actin mRNA level (× 10^–4^, mean ± SEM). (**B**) Effects of co-culture of OA patients’ synovium-derived, cultured MCs (n = 5) with RA patients’ fibroblasts (n = 2) on expression levels of mRNA for PTGS1, PTGS2, TBXAS1 and LTC4S in the OA patients’ MCs. The data are shown as the ratio of the enzyme mRNA level in the MCs alone to that in the MCs after co-culture with the RA patients’ fibroblasts (mean ± SEM).

The SCF–Kit system alone is insufficient to fully drive the maturation of MCs, since culture of immature MCs with fibroblasts, but not with SCF alone, can induce differentiation into mature MCs^28^. We reported that group III phospholipase A2 secreted from MCs couples with fibroblastic LPGDS to form PGD_2_, which facilitates MC maturation via PGD_2_ receptor DP^29^. Thus, we hypothesized that RA patients’ fibroblasts might influence the phenotypes of MCs. We first compared the expression levels of mRNAs for PTGS1, PTGS2, TBXAS1 and LTC4S between the RA patients’ and OA patients’ synovial fibroblasts. No significant differences were found (Fig. 4A). Next, synovium-derived, cultured MCs from OA patients were cultured with and without RA patients’ fibroblasts for 96 h. The MCs were then collected, and the expression levels of mRNAs for PTGS1, PTGS2, TBXAS1 and LTC4S were compared between the MCs cultured with and without the fibroblasts. No differences in the expression levels were found (Fig. 4B).

### Relationship between expression levels of miR-199a-3p and PTGS2 mRNA in synovium-derived, cultured MCs from OA and RA patients

**Figure 5:**
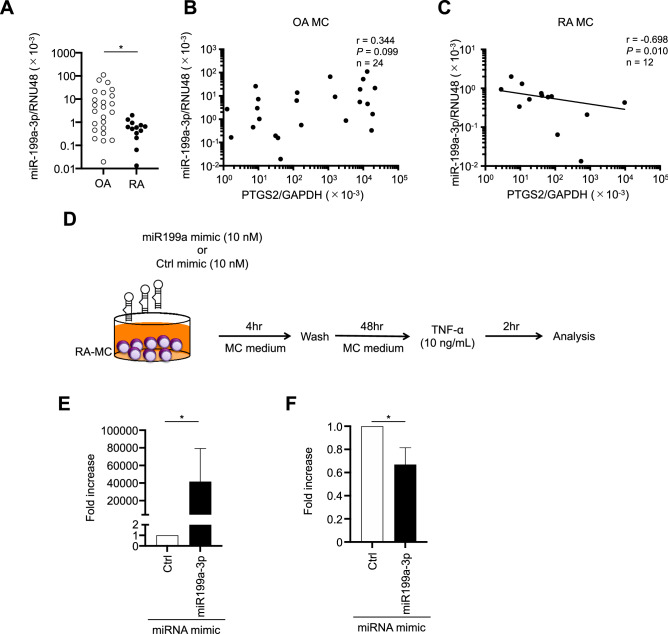
Relationship between miR-199a-3p expression levels and PTGS2 mRNA expression levels in synovium-derived, cultured MCs from RA and OA patients. (**A**) Comparison of miR-199a-3p expression levels in synovium-derived, cultured MCs obtained from OA (n = 24) and RA (n = 12) patients. The data are shown as the ratio of the miR-199a-3p level to the RNU48 level (× 10^–3^, mean ± SEM). Significance was determined using the Mann–Whitney *U* test. (**B**,**C**) Plots of expression level of miR-199a-3p/RNU48 vs. expression level of PTGS2/GAPDH in synovium-derived, cultured MCs obtained from OA (n = 24, **B**) and RA (n = 12, **C**) patients. Each point represents one donor. (**D**) Illustration of procedure for transduction of miRNA mimic to synovium-derived, cultured MCs obtained from RA patients and activation of the cells (**E**). Expression levels of miR-199a-3p mRNA in control miRNA mimic-transduced (Ctrl) and miR-199a-3p mimic-transduced synovium-derived, cultured MCs obtained from RA patients (miR-199a-3p). The expression level of miR-199a-3p in control miRNA mimic-transduced cells was designated as 1. The results are the mean ± SEM for three different experiments. (**F**) Expression levels of PTGS2 mRNA in control miRNA mimic-transduced (Ctrl), and miR199a-3p mimic-transduced synovium-derived, cultured MCs obtained from RA patients (miR-199a-3p) in response to 10 ng/mL of TNF-α for 2 h. The expression level of PTGS2 mRNA in control miRNA mimic-transduced cells was designated as 1. The results are the mean ± SEM for three different experiments. *indicates *P* < .05.

micro RNAs (miRNAs) are noncoding RNAs implicated in the regulation of gene expression underlying many relevant physiological processes, including cell activation. We investigated one mechanism causing different mRNA expression levels for prostanoid synthetases between RA and OA patients’ synovial MCs. That is, we compared miRNA expression profiles using microRNA array. Thirty miRNAs showed expression levels that were significantly more than three times higher in OA patients’ MCs compared with RA patients’ MCs (Table 1). Among those 30 miRNAs, miR-199a-3p was reported to down-regulate PTGS2 mRNA expression in OA chondrocytes^30,31^, cultured human fetal lung epithelial cells^32^, cultured human myometrial cells^33^ and human endometrial surface epithelial cells^34^. Thus, we performed quantitative RT–PCR to compare the miR-199a-3p expression levels between synovial MCs from RA and OA patients. The results showed that the miR-199a-3p expression level was significantly higher in the OA patients than in the RA patients (*P* < 0.05, Fig. 5A). Moreover, that expression level correlated inversely with the PTGS2 mRNA expression level in the RA patients’ MCs (r = − 0.698, *P* = 0.010; Fig. 5C), but not in the OA patients’ MCs (Fig. 5B). Tables 2 and 3 shows the characteristics of the patients with OA and RA, respectively.

**Table 1:** List of genes whose expression levels of miRNAs were significantly more than three times higher in MCs from OA patients than in MCs from RA patients.

Systematic_name	Fold increase ([RA] vs [OA])	Regulation ([RA] vs [OA])	[OA] (raw)	[RA] (raw)	[OA] (normalized)	[RA] (normalized)
ebv-miR-BART12	− 69.01	Down	5.22	0.10	− 1.64	− 7.74
hsa-miR-4254	− 5.50	Down	1.87	0.10	− 5.28	− 7.74
hsa-miR-564	− 5.34	Down	1.71	0.10	− 5.33	− 7.74
hsv2-miR-H22	− 5.27	Down	1.65	0.10	− 5.35	− 7.74
hsa-miR-193b	− 4.41	Down	0.99	0.10	− 5.60	− 7.74
hsa-miR-498	− 4.35	Down	0.96	0.10	− 5.62	− 7.74
hsa-miR-718	− 4.24	Down	0.89	0.10	− 5.66	− 7.74
hsa-miR-4327	− 4.21	Down	8.03	8.61	− 1.00	− 3.08
hsa-miR-127-3p	− 4.18	Down	0.86	0.10	− 5.68	− 7.74
hsa-miR-342-5p	− 4.14	Down	0.83	0.10	− 5.69	− 7.74
hsa-miR-3149	− 4.12	Down	0.82	0.10	− 5.70	− 7.74
hsv1-miR-H8	− 4.04	Down	0.78	0.10	− 5.73	− 7.74
hsa-miR-3117	− 3.95	Down	5.35	5.02	− 1.62	− 3.61
hsa-miR-199a-3p	− 3.67	Down	18.34	30.16	− 0.27	− 2.14
hsa-miR-34a*	− 3.44	Down	1.65	1.52	− 4.15	− 5.93
hsv1-miR-H6-5p	− 3.34	Down	2.43	2.31	− 2.61	− 4.35
hsa-miR-664*	− 3.28	Down	2.96	3.79	− 2.39	− 4.10
hsa-miR-502-3p	− 3.25	Down	3.87	4.80	− 1.94	− 3.64
hcmv-miR-US4	− 3.23	Down	0.43	0.10	− 6.05	− 7.74
hsa-miR-1288	− 3.20	Down	4.03	4.72	− 2.00	− 3.68

*Raw* data from miRNA microarrays, *normalized* normalized data obtained from miRNA microarrays (please see “Materials and methods” section), *MCs* mast cells, *OA* osteoarthritis, *RA* rheumatoid arthritis.

**Table 2:** Characteristics of patients with OA.

Patient	Sex	Age (y)	Disease duration (y)	WBC (/mm^3^)	CRP (mg/dl)	Anti-CCP Ab (U/ml)	MMP3 (ng/ml)	RF (mg/dl)	Treatment
OA1	F	62	4	6200	< 0.1	n.d	n.d	n.d	NSAIDs
OA2	F	83	9	2700	0.13	n.d	n.d	n.d	NSAIDs
OA3	F	65	3	7300	< 0.1	n.d	n.d	n.d	NSAIDs
OA4	F	81	10	7500	< 0.1	n.d	n.d	n.d	NSAIDs
OA5	F	61	11	6300	0.18	n.d	n.d	n.d	NSAIDs
OA6	M	80	8	6800	< 0.1	n.d	n.d	n.d	NSAIDS
OA7	F	67	7	6800	< 0.1	n.d	n.d	n.d	NSAIDs
OA8	F	81	10	7500	< 0.1	n.d	n.d	n.d	NSAIDs
OA9	M	71	2	6500	< 0.1	n.d	n.d	n.d	NSAIDs
OA10	F	63	10	5300	< 0.1	n.d	n.d	n.d	NSAIDs
OA11	F	75	8	4500	< 0.1	n.d	n.d	n.d	NSAIDs
OA12	F	80	15	7900	< 0.1	n.d	n.d	n.d	NSAIDs
OA13	F	66	4	6700	< 0.1	n.d	n.d	n.d	NSAIDs
OA14	F	85	20	6300	0.5	n.d	n.d	n.d	NSAIDs
OA15	F	78	11	4700	0.1	n.d	n.d	n.d	Tramadol + Acetaminophen
OA16	M	77	1	5400	0.1	n.d	n.d	n.d	None
OA17	F	83	2	4400	0.1	n.d	n.d	n.d	Acetaminophen
OA18	F	77	4	6400	0.1	n.d	n.d	n.d	None
OA19	F	76	30	4700	0.1	n.d	n.d	n.d	None
OA20	M	77	6	5300	0.1	n.d	n.d	n.d	NSAIDs
OA21	F	74	5	6100	0.1	n.d	n.d	n.d	NSAIDs
OA22	F	71	3	5600	0.1	n.d	n.d	n.d	NSAIDs
OA23	F	69	2	6900	0.97	n.d	n.d	n.d	None
OA24	F	77	6	7300	1.66	n.d	n.d	n.d	None

*anti-CCP Ab* anti-cyclic citrullinated peptide antibody, *CRP* C-reactive protein, *F* female, *M* male, *MMP3* matrix metalloproteinase 3, *n.d* not done, *NSAIDs* non-steroidal anti-inflammatory drugs, *OA* osteoarthritis, *RF* rheumatoid factor, *WBCs* white blood cells.

**Table 3:** Characteristics of patients with RA.

Patient	Sex	Age (y)	Disease duration (y)	WBC (/mm^3^)	CRP (mg/dl)	Anti-CCP Ab (U/ml)	MMP3 (ng/ml)	RF (mg/dl)	Treatment
RA1	F	74	30	7000	0.28	347	152	49	NSAIDs + DMARDs
RA2	M	69	4	7900	3.4	875	788	69	MTX + anti-TNF-α
RA3	M	69	4	4000	2.9	875	788	69	MTX + anti-TNF-α
RA4	M	76	10	5700	2.96	7.9	530	380	MTX + anti-IL6
RA5	F	78	10	8000	3.78	727	386.7	n.d	MTX
RA6	F	76	15	4600	0.43	20.7	133.3	15.8	MTX
RA7	M	67	17	8000	0.73	570	256	78	MTX + DMARDs
RA8	F	71	15	7300	0.39	12.1	102.7	86	MTX
RA9	F	70	30	5600	0.14	n.d	86.8	7.9	DMARDs
RA10	F	75	25	3100	0.54	0.6	87	4.7	MTX + DMARDs
RA11	F	85	6	9200	2.13	80	190	228.2	MTX + PSL
RA12	F	74	10	5300	0.31	860	59.9	240	MTX

*anti-CCP Ab* anti-cyclic citrullinated peptide antibody, *anti-IL6* anti-interleukin 6 antibody therapy, *anti-TNF-α* anti-tumor necrosis factor-α antibody therapy, *CRP* C-reactive protein, *DMARDs* disease modifying antirheumatic drugs, *F* female, *M* male, *MMP3* matrix metalloproteinase 3, *MTX* methotrexate, *n.d* not done, *NSAIDs* non-steroidal anti-inflammatory drugs, *PSL* prednisolone, *RA* rheumatoid arthritis, *RF* rheumatoid factor, *WBC* white blood cells.

To analyze the effect of miR-199a-3p on PTGS2 expression, we overexpressed miR-199a-3p in synovium-derived, cultured MCs obtained from RA patients by a transduction of miRNA mimics. Figure 5D depicts the method for transduction of miRNA mimic to synovium-derived, cultured MCs obtained from RA patients and activation of the cells. Briefly, miR199a-3p or control miRNA mimics were transduced to the cells, and then the cells were activated with TNF-α. In Fig. 5E, the miR199a-3p expression level was significantly increased in synovium-derived, cultured MCs obtained from RA patients transduced with an miR199a-3p mimic compared with control miRNA mimic (*P* < 0.05, Fig. 5E). We stimulated these MCs with TNF-α (10 ng/mL) for 2 h and analyzed PTGS2 expression in control miRNA mimic–transduced and miR199a-3p mimic–transduced cells. The PTGS2 expression in miR199a-3p mimic-transduced cells was significantly lower than in control miRNA mimic-transduced cells after 2 h of stimulation with TNF-α (*P* < 0.05, Fig. 5F).

### Comparison of PGD_2_ and PGE_2_ concentrations in SF obtained from OA and RA patients

**Figure 6:**
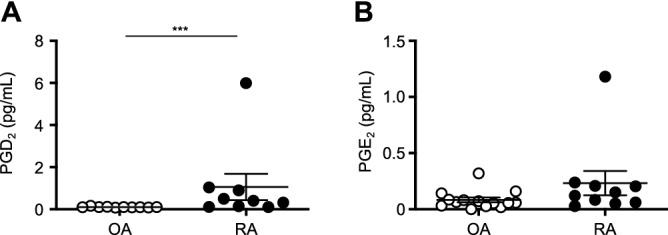
Comparison of PGD_2_ and PGE_2_ concentrations in SF obtained from OA and RA patients. SF was obtained from OA (n = 14) and RA (n = 10) patients. Each point represents one donor. Significance was determined using the Mann–Whitney *U* test (***P < .0005).

We performed EIA to measure the PGD_2_ and PGE_2_ concentrations in SF obtained from OA (n = 14) and RA (n = 10) patients. Tables 2 and 3 show the characteristics of the patients. The concentration of PGD_2_ (Fig. 6A), but not PGE_2_ (Fig. 6B), was significantly higher in the RA patients’ SF than in the OA patients’ SF.

## Discussion

We present the first evidence that synovial MCs from RA and OA patients have different phenotypes and function. FcRγ aggregation induced significantly higher PGD_2_ production by the synovium-derived, cultured MCs from RA patients via miR-199a-3p/PTGS2 axis compared with the OA patients’ MCs.

Since the LTC4S expression level showed the tendency of being higher in the RA patients’ MCs than in the OA patients’ MCs (Fig. 1E), LTC_4_ production by the RA patients’ MCs following FcεRI aggregation was significantly higher (Fig. 2G). In contrast, LTB_4_ production by the RA patients’ MCs following FcεRI aggregation was significantly lower than that by the OA patients’ MCs (Fig. 2H). Since LTB_4_ and LTC_4_ are metabolites of LTA_4_, LTB_4_ synthesis in RA patients’ MCs may be relatively lower. LTB_4_ and LTC_4_ by MCs from both RA and OA patients after FcγRI aggregation was markedly lower than after FcεRI aggregation (Fig. 2C,D,G,H). FcεRI consists of heterotetramer αβγ_2_-chains. On the other hand, FcγRI consists of heterotrimer αγ_2_-chains. Since FcεRI β-chain reportedly amplified LT production by mouse MCs^35^, FcγRI aggregation might cause no or lesser amounts of LT production by human MCs as well. Thus, immune complexes might induce large amounts of only PGD_2_ among the arachidonic acid metabolites in RA patients’ synovial MCs.

We previously reported that co-culture of IL-3-dependent mouse bone marrow-derived MCs with mouse fibroblasts resulted in 1.4-fold and 387-fold up-regulation of PTGS1 and PTGS2 mRNA expression, respectively, in the MCs^29^. Thus, we hypothesized that fibroblasts from RA patients might influence PTGS1 and PTGS2 mRNA expression in OA patients’ MCs, but our co-culture experiment did not find that to be true. The reason is not clear, but it might be because IL-3-dependent mouse bone marrow-derived MCs are immature MCs, whereas human synovium-derived, cultured MCs are fully mature MCs^7^.

All of our OA patients and some RA patients had been treated with NSAIDs. NSAIDs inhibit PTGS enzymes^36^. However, NSAIDs do not affect PTGS1 and PTGS2 mRNA expression. Synovium-derived, cultured MCs from both OA and RA patients had been cultured under the same conditions for more than 12 weeks. Therefore, it would be difficult to conclude that NSAID treatment of patients might affect the gene expression profiles of the cultured MCs derived from synovia of OA and RA patients. We thus hypothesized that epigenetic modification might be the cause of the difference in the gene expression profiles of MCs derived from synovia of OA and RA patients.

Microarray-based screening found that the expression levels of thirty miRNAs were significantly more than three times higher in OA patients’ MCs than in RA patients’ MCs (Table 1). Among those 30 miRNAs, miR-199a-3p is a direct regulator of PTGS2 expression in OA chondrocytes^30,31^, cultured human fetal lung epithelial cells^32^, cultured human myometrial cells^33^ and human endometrial surface epithelial cells^34^. The miR-199a-3p/PTGS2 axis plays different roles in different cells and diseases. In human OA chondrocytes, miR-199a-3p directly suppressed the luciferase activity of a PGST2 3′UTR reporter construct and inhibited IL-β-induced PTGS2 protein, suggesting that miR-199a-3p may be an important regulator of human cartilage hemostasis and a new target for OA therapy^30^. In fact, epigallocatechin-3-*O*-gallate, the most abundant and active polyphenol in green tea, which has been reported to have anti-arthritic effects, inhibited PTGS2 mRNA/protein expression and PGE_2_ production by up-regulating miR-199a-3p expression in IL-1β-stimulated human OA chondrocytes^31^. The developmental decline in miR-199a/miR-214 expression in the fetal lung led to increased expression of critical targets, including PTGS2, NF-κB p50/p65, CREB1 and C/EBPβ that enhance surfactant protein-A expression and alveolar type II cell differentiation^32^. The levels of the clustered miRNAs, i.e., miR-199a-3p and miR-214, were significantly decreased in the myometrium of pregnant mice and humans, whereas the miR-199a-3p/miR-214 target, PTGS2, which induced synthesis of contracting PGs, was coordinately increased^33,37^. Overexpression of miR-199a-3p and miR-214 in cultured human myometrial cells inhibited PTGS2 protein and blocked TNF-α-induced myometrial cell contractility, suggesting their physiological relevance^33^. Epithelial sodium channel-dependent CREB activation led to suppression of miR-199a-3p and miR101, which in turn augmented PTGS2 up-regulation during embryo implantation^34^. We found that miR-199a-3p correlated inversely with PTGS2 expression in RA patients’ MCs (r = − 0.698, *P* = 0.010; Fig. 5C), suggesting that miR-199a-3p may be a regulator of PTGS2 mRNA expression in RA patients’ synovial MCs. Furthermore, we confirmed that PTGS2 mRNA expression by miR199a-3p mimic-transduced synovium-derived, cultured MCs obtained from RA patients was significantly lower than that by control miRNA mimic-transduced cells, when the cells were activated with TNF-α (*P* < 0.05, Fig. 5F). The 3′ UTR of the mTOR gene is reportedly targeted by miR-498, which was included in the list of thirty genes. Consequently, silencing of mTOR reduced PTGS2 expression in OA chondrocytes^38^. Thus, other miRNAs might also affect PTGS2 mRNA expression in RA patients’ synovial MCs.

PGD_2_ was reported to be detected in SF obtained from RA patients^11,22,39^. PGD_2_ is synthesized by various cells, including MCs^16^, antigen-presenting cells^17^, T helper 2 (Th2) lymphocytes^18^ and synovial fibroblasts^40^. The cell sources of PGD_2_ in RA patients’ SF have not been identified. In our study, OA and RA patients’ fibroblasts showed no significant differences in expression of PTGS1 and PTGS2 mRNAs. Thus, increased PGD_2_ synthesis would not be due to production by synovial fibroblasts. Our in vitro study found that synovial MCs (10^5^ cells) from RA patients produced 4000 pg/mL PGD_2_ following FcγRI aggregation. Furthermore, PGD_2_ metabolites have been reported to be biomarkers of in vivo MC activation in RA patients^41^. Therefore, MCs might be one cell source of PGD_2_ in RA patients’ SF.

Upregulation of the PTGS1 and PTGS2 pathways of arachidonic acid (AA) is thought to be involved in the development of rheumatic diseases, and targeting these pathways might lead to improved treatment strategies^11^. Thus, to clarify the quantitative and qualitative changes in lipid mediators in the synovium of severe RA patients, we recently compared the profiles of lipid mediators in SF obtained from RA and OA patients using liquid chromatography-tandem mass spectrometry/mass spectrometry^42^. The concentrations (levels based on the area-under-the-curve/mL) of the majority of PTGS-1/2 products of AA appeared to be higher in SF from RA patients compared with OA patients. We determined the absolute concentrations (pmol/mL) of representative eicosanoids, including 6-keto PGF_1α_ (a stable metabolite of PGI_2_), PGF_2α_, PGE_2_, PGD_2_ and 12-hydroxyheptadecatrienoic acid (HHT), in the SF from RA and OA patients. The PGF_2α_ and PGE_2_ concentrations were significantly higher in the RA patients’ SF. Thus, although the PGD_2_ concentration in the SF did not differ significantly between the RA and OA patients in our previous study^42^, we confirmed our earlier findings regarding upregulation of the PTGS pathways in RA compared with in OA^42^.

This study has a number of limitations. First, all the samples used in this study were obtained from hospitalized patients who had undergone total knee replacement surgery. It was reported that RA patients in remission had significantly reduced synovial MC density compared with patients with clinically active RA^43^. Thus, the enrollees were limited to patients with severe clinical disease, hence limiting extrapolation of the findings to patients with milder clinical disease. Second, we cannot rule out the possibility that use of a wide range of antirheumatic drugs-including methotrexate, oral glucocorticoids, anti-TNF-α therapy and anti-IL-6 therapy that potently suppress specific inflammation, might be responsible for the differences in gene expression profiles in OA and RA patients’ MCs. However, the RA patients’ anti-TNF-α therapy and anti-IL-6 therapy had been discontinued 2–4 weeks before the total knee arthroplasty. Since expression of PTGS2 mRNA and protein is reportedly enhanced in various human cell types by such inflammatory cytokines as IL-1β and TNF-α^44^, anti-TNF-α therapy and anti-IL-6 therapy would not enhance PTGS2 mRNA expression levels.

PGD_2_ was reported to be active in the resolution of inflammation in experimentally induced arthritis in mice^19,21^ and in human RA^22^. Increased PGD_2_ may trigger an anti-inflammatory/pro-resolution cascade. That is because it spontaneously undergoes non-enzymatic dehydration and is converted into 15-deoxy-Δ12,14-prostaglandin J_2_ (15d-PGJ_2_). 15d-PGJ_2_ is a cyclopentenone PG that has been shown to be immuno-modulatory and anti-inflammatory due to its ability to inhibit NFκB signaling and cytokine release and to act as an agonist of PPARγ^45^. However, the role of MC-derived PGD_2_ in the pathogenesis of RA should be determined in MC-deficient experimental arthritis in mice reconstituted with bone marrow-derived MCs obtained from HPGDS-deficient mice. NSAIDs’ inhibition of cyclooxygenase-dependent PG synthesis ameliorates RA manifestation because they inhibit mainly production of PGE_2_, which exacerbates synovial inflammation in RA patients^46^.

Since ILC2s were reportedly involved in the pathogenesis of RA^23,24^, we investigated the effect of PGD_2_ on RA-related cytokine production (IL-6, IL-8, IL-9 and TNF-α) by ILC2s. We found that PGD_2_ induced IL-8 production by ILC2s, suggesting that PGD_2_-producing MCs induce neutrophil recruitment into the synovium of RA patients.

In conclusion, we demonstrated that human synovial MCs might regulate inflammation through hyper-production of PGD_2_ in RA following FcRγ aggregation. These findings indicate that human MCs show functional heterogeneity among diseases.

## Materials and methods

### Patient enrollment and processing of SF

We enrolled RA and OA patients. The diagnosis of each patient was established by the treating doctor. SF and synovial tissue samples were obtained during total knee arthroplasty performed at the Department of Orthopeadic Surgery, Nihon University, after receiving informed consent. Two milliliters of SF were treated with hyaluronidase, followed by centrifugation at 860×*g* for 10 min. The supernatants were collected, and the tubes were filled with N_2_ gas. The samples were then frozen at − 80 °C.

### Reagents

Human IgE was purchased from Calbiochem (San Diego, CA). Anti-FcεRIα monoclonal antibodies (mAbs) (clone AER-37) and anti-Kit mAb (clone YB5.B8) were purchased from Biolegend (San Diego, CA) and BD Biosciences (Franklin Lakes, NJ, USA), respectively.

### Purification of dispersed synovial MCs and generation of synovium-derived, cultured MCs

Human synovial MCs were purified with anti-FcεRIα and anti-Kit mAbs using FACS Aria IIu (BD Biosciences). The purities of MCs were > 99%. Human synovium-derived cultured MCs were generated as described previously^8^. Briefly, fresh samples of synovial tissues were obtained after total knee arthroplasty at Nihon University, after obtaining informed consent. Treatment of RA patients with anti-TNF-α therapy and anti-IL-6 therapy had been discontinued 2–4 weeks before total knee arthroplasty, in accordance with the Japanese Guidelines for the use of Infliximab and Etanercept in RA^47^. Briefly, synovial cells were enzymatically dispersed and centrifuged using a density-gradient consisting of 22.5% HistoDenz solution (Sigma-Aldrich; St. Louis, MO, USA) and lymphocyte separation medium (LSM; MP Biomedicals; Santa Ana, CA, USA). Cells at the LSM interface and in the pellet fraction were collected and washed. The cells were then cultured in serum-free Iscove’s methylcellulose medium (Stem Cell Technologies Inc.; Vancouver, BC, Canada) and Iscove’s Modified Dulbecco’s Medium (IMDM; Thermo Fisher Scientific; Waltham, MA, USA) supplemented with 200 ng/mL recombinant human stem cell factor (rhSCF) (PeproTech; Rocky Hill, NJ, USA) and 50 ng/mL rhIL-6 (PeproTech). On day 42, methylcellulose was dissolved in PBS, and the cells were resuspended and cultured in IMDM containing 0.1% BSA, 100 ng/mL rhSCF and 50 ng/mL rhIL-6 (designated as MC medium).

### Synovial fibroblasts

Fresh samples of synovial tissues were obtained after total knee arthroplasty at Nihon University, after obtaining informed consent. Synovial fibroblasts were obtained after culturing enzymatically-dispersed synovial cells^7^.

### Isolation and expansion of human group 2 innate lymphoid cells (ILC2s)

Peripheral blood mononuclear cells (PBMCs) were isolated from whole blood of healthy volunteers by centrifugation using LSM. Lineage-negative cells were enriched from the isolated PBMCs using magnetic-activated cell sorting (MACS) and Microbeads (Miltenyi Biotec; GladBach, Germany) in accordance with the manufacturer’s protocol, with some modification. Briefly, 5 × 10^7^ to 1 × 10^8^ cells were suspended in 200 μL of cold MACS buffer (PBS containing 2% fetal bovine serum [FBS; Thermo Fisher Scientific] and 2 mM EDTA [Eastman Kodak Company; Rochester, NY, USA]) and incubated for 30 min at 4 °C with 50 μL of FcR blocking reagent (Miltenyi Biotec) and 25 μL of anti-human CD3, CD4, CD8, CD11b, CD14, CD16 and CD19 Microbeads (Miltenyi Biotec). The lineage-negative cells were washed with MACS buffer, centrifuged at 490×*g* for 5 min, resuspended in 500 μL of cold MACS buffer and applied to a MACS CS Column (Miltenyi Biotec) on a VarioMACS Separator (Miltenyi Biotec). The flow-through was collected as the lineage-negative fraction. The column was then washed 3 times with cold MACS buffer. The collected cells were treated for 10 min at room temperature with a Zombie NIR Fixable Viability Kit (Biolegend; San Diego, CA, USA) to label dead cells. The treated cells were then washed with PBS and incubated with Human TruStain FcX (Biolegend) for 15 min at room temperature to block non-specific binding. After washing with MACS buffer, the cells were stained for 30 min at 4 °C with FITC-Lineage Cocktail-1 (BD Biosciences; San Jose, CA, USA), PerCP-Cy5.5-anti-human CD45 mAb (clone HI30; BD Biosciences), PE-anti-human CD161 mAb (clone HP-3G10; Biolegend) and Brilliant Violet 421-conjugated anti-human CRTh2 mAb (clone BM16; BD Biosciences). Lin^-^ CD45^+^ CD161^+^ CRTh2^+^ cells were isolated with FACSAria IIu (BD Biosciences). The sorted cells (designated as ILC2s; 2000 cells per well) were cultured in the presence of mitomycin C-treated PBMCs (2 × 10^5^ cells per well) as feeder cells in 96-well U-bottom plates (CORNING; Corning, NY, USA) in Yssel’s medium (IMDM supplemented with 1% heat-inactivated human AB serum; Access Biologicals; Vista, CA, USA) and 100 IU/mL Teceleukin (rhIL-2; Kyowa Pharmaceutical Industry; Osaka, Japan). After 3 weeks of in vitro expansion the ILC2 number reached approximately 5 × 10^6^ to 1 × 10^7^ cells, which were used in subsequent experiments. ILC2s were stimulated with PGD_2_ (0–100 nM) for 24 h and the cytokine levels in the cell supernatants were measured by enzyme-linked immunosorbent assays (ELISAs).

### RNA isolation and RT-PCR

Total RNA was isolated from the synovium-derived, cultured MCs by using a miRNeasy Micro kit (Qiagen, Hilden, Germany) and then quantified using NanoDrop ND-1000 (Thermo Fisher Scientific) according to the manufacturer’s instructions. Real-time quantitative RT-PCR was performed as described previously^8^. Human gene-specific primers and probe sets for PTGS1, PTGS2, PTGES, LTC4S, LTC4S, TBXAS1, HPGDS, GAPDH, miR-199a-3p and RNU48 were designed using the Assay-on-Demand service (Thermo Fisher Scientific).

### Microarray experiments and data analysis

Synovium-derived, cultured MCs from 3 RA patients and 3 OA patients were screened for mRNAs and miRNAs using Agilent SurePrint G3 Human GE 8 × 60 k Microarray Kit and Agilent SurePrint G3 Human microRNA (miRNA) 8 × 60 k Microarray Kit (Agilent Technologies, Santa Clara, CA), respectively. One × 10^4^ MCs were used in the microarray experiments. Data analysis was performed with GeneSpring12.5 (Agilent Technologies; Santa Clara, CA). To adjust for variation in the staining intensity between microarrays, the expression levels of all the genes on a given microarray chip were normalized by dividing the measurement by the 75th percentile of all measurements on that microarray. Quality control was achieved with the Filter Probe sets by Flags tool and, as result, 32,425 of 42,545 probes (mRNA) and 405 of 1368 probes (miRNA) were used for further analyses. mRNAs or miRNAs showing significant signal intensity (> 2.0-fold, adjusted *P* < 0.05; Mann–Whitney *U*-test with Benjamini–Hochberg false discovery rate correction) were determined to be differentially expressed (up- or down-regulated). The false discovery rate is a comparison of the number of times that the real data has a certain *P* value versus the number of times that randomized data has the same or better *P* value. The false discovery rate addresses the multiple comparisons problem that occurs when calculating *P* values for hundreds or thousands of categories, and protects against over-interpreting *P* values that do not have biological meaning^48^. Hierarchical clustering was performed on the basis of the gene expression data. Of the differentially expressed miRNAs, target miRNAs that interact with target genes were determined by a search of the literature. To further investigate the global molecular network, especially to identify upstream cytokines in pathological signaling cascades, the target genes were imported into IPA (version 27821452)^49^.

### Ethical approval

This study was approved by the Ethics Committee of the Nihon University School of Medicine (RK-160112-2) and the Ethics Committee of National Research Center for Child Health and Development (approval number: 476). All the subjects provided written informed consent in accordance with the Helsinki Declaration of the World Medical Association.

### Transduction of miRNA mimics

MISSION microRNA Mimics Negative Control 2 (HMC0003) and mimics for miR199a-3p (HMI0340) were purchased from Sigma-Aldrich. These small RNAs were transduced into synovium-derived, cultured MCs obtained from RA patients using MISSION siRNA Transfection Reagent (Sigma-Aldrich) and Nucleic Acid Transfection Enhancer (NATE, InvivoGen, San Diego, CA, USA) in accordance with the manufacturer’s instructions. Four hours later, the cells were washed with PBS to remove excess complexes, and the transduced cells were cultured with MC medium. Synovium-derived, cultured MCs obtained from RA patients transduced with control miRNA mimic or miR199a-3p mimic were activated with recombinant human TNF-α (R&D Systems, Minneapolis, MN, USA). Then the expression level of PTGS2 in these cells was analyzed by quantitative RT-PCR.

### MC activation

For aggregation of FcεRI, MCs were sensitized with 0.5 μg/ml human IgE (Merck Millipore, Burlington, MA, USA) for 30 min at 37 °C. The cells were then washed once and resuspended in Hepes buffer or MC medium. The IgE-sensitized MCs were stimulated with polyclonal rabbit anti-human IgE (Agilent) for 30 min for the histamine and lipid mediator assays (2 × 10^3^ MCs/100 μl). For aggregation of FcγRI, the MCs were incubated with 1 or 10 μg/ml of F(ab′)_2_ fragments of anti-human FcγRI (F(ab′)_2_αFcγRI clone 10.1), or mouse F(ab′)_2_ fragments of IgG1 (F(ab′)_2_mIgG1; Jackson ImmunoResearch Laboratories) for 30 min at 37 °C. The cells were washed once and resuspended in Hepes buffer or MC medium. FcγRI was cross-linked by incubating the MCs with the indicated concentrations of goat F(ab’)_2_ fragments of anti-mouse F(ab′)_2_ fragments of IgG (gF(ab′)_2_αmF(ab′)_2_; Jackson ImmunoResearch Laboratories) for 30 min for the histamine and lipid mediator assays (2 × 10^3^ MCs/100 μL). Independent experiments were performed using MCs from different donors.

### Mediator assays

Histamine was measured using an enzyme immunoassay (EIA) kit (Bertin Pharma; Montigny le Bretonneux, France). PGD_2_ was measured using a Prostaglandin D_2_-MOX EIA kit (Cayman Chemical Company; Ann Arbor, MI). LTC_4_, LTB_4_ and PGE_2_ were measured using EIA kits (R&D Systems; Minneapolis, MN). IL-6, IL-8 and TNF-α were also measured using ELISA kits (Biolegend). IL-9 was measured using a Human IL-9 Uncoated ELISA kit (Thermo Fisher Scientific). The percent histamine release was calculated as follows: (histamine released/total histamine content of unstimulated MCs) × 100%.

### Co-culture of synovial MCs and fibroblasts

Synovial fibroblasts from RA patients were cultured in IMDM supplemented with 2% FBS for 48 h, and the fibroblasts were grown to confluence. Human synovium–derived, cultured MCs from OA patients were overlaid on the fibroblasts and cultured in IMDM containing 0.1% BSA, 100 ng/mL rhSCF and 50 ng/mL rhIL-6 for 96 h. The MCs were then purified with anti-FcεRIα and anti-Kit mAbs using FACS Aria IIu (BD Biosciences).

### Statistical analysis

To evaluate the quantitative variables, the Mann–Whitney *U* test was used because of nonparametric distribution of the data. Figures 2 and 3 were analyzed by Sidak’s–Bonferroni method. Spearman rank correlation coefficients were calculated to determine the strength of correlations between continuous variables. *P* values were considered significant at *P* < 0.05.
